# A high-throughput pipeline for design and selection of peptides targeting the SARS-Cov-2 Spike protein

**DOI:** 10.1038/s41598-021-01225-2

**Published:** 2021-11-05

**Authors:** Monica Wolfe, Sean Webb, Yaroslav Chushak, Rachel Krabacher, Yi Liu, Nathan Swami, Svetlana Harbaugh, Jorge Chávez

**Affiliations:** 1 711th Human Performance Wing, Air Force Research Laboratory, Wright-Patterson Air Force Base, Dayton, OH 45433 USA; 2 UES, Inc., Dayton, OH 45432 USA; 3 Henry M. Jackson Foundation, Dayton, OH 45433 USA; 4 Department of Electrical and Computer Engineering, University of Virginia, Charlottesville, VA 22904 USA; 5 Present Address: Materials & Manufacturing Directorate, Air Force Research Laboratory, Wright-Patterson Air Force Base, Dayton, OH 45433 USA

**Keywords:** Assay systems, Diagnosis

## Abstract

Rapid design, screening, and characterization of biorecognition elements (BREs) is essential for the development of diagnostic tests and antiviral therapeutics needed to combat the spread of viruses such as severe acute respiratory syndrome coronavirus 2 (SARS-CoV-2). To address this need, we developed a high-throughput pipeline combining in silico design of a peptide library specific for SARS-CoV-2 spike (S) protein and microarray screening to identify binding sequences. Our optimized microarray platform allowed the simultaneous screening of ~ 2.5 k peptides and rapid identification of binding sequences resulting in selection of four peptides with nanomolar affinity to the SARS-CoV-2 S protein. Finally, we demonstrated the successful integration of one of the top peptides into an electrochemical sensor with a clinically relevant limit of detection for S protein in spiked saliva. Our results demonstrate the utility of this novel pipeline for the selection of peptide BREs in response to the SARS-CoV-2 pandemic, and the broader application of such a platform in response to future viral threats.

## Introduction

Since its discovery in late 2019, severe acute respiratory syndrome coronavirus 2 (SARS-CoV-2) has grown into a worldwide pandemic, due largely in part to a high rate of transmission (with R_0_ estimates as high as 5.7^1^) and asymptomatic presentation (current estimates report 17% of cases are asymptomatic^2,3^). Delays in the development of reliable diagnostic tests, especially for point-of-care detection of acute infection, have made controlling the spread of the virus difficult, further contributing to the severity of the pandemic. Currently, molecular assays such as reverse transcription polymerase chain reaction (RT-PCR) are the gold-standard for identification of acute stage infection, however these techniques require specialized laboratory instruments and lengthy experimental procedures with slow turn-around time^4^. Antigen tests are able to yield results quicker than RT-PCR tests, however, issues with sensitivity limit their utility in the asymptomatic population^5^.

SARS-CoV-2 is the third coronavirus (CoV) in the past two decades to cause severe illness in humans. Consistent with other CoVs, the virus consists of a single positive strand RNA genome that encodes four structural proteins: spike (S), envelope (E), matrix (M), and nucleocapsid (N). Of these, the S protein, which consists of the S1 and S2 subunits, has been identified as the key structural protein responsible for initiating host cell attachment and membrane fusion^6,7^. SARS-CoV-2 entry into host cells is facilitated by the S protein’s receptor binding domain (RBD) located on the S1 subunit, which binds to the angiotensin-converting enzyme 2 (ACE2) receptor located on epithelial cells lining the respiratory tract^8^.

Viral surface proteins, such as the S protein, are promising targets for both virus detection and neutralization. Early in the pandemic, the crystal structure of ACE2 and SARS-CoV-2 RBD was solved (PDB code: 6M17^6^) and used to identify a region within the α1 helix of the ACE2 protein directly involved in binding to the S protein RBD^9–11^. Molecular dynamics simulations demonstrated that a short 23-mer peptide fragment from ACE2 (named SBP1) could bind to the SARS-CoV-2 RBD with micromolar affinity (K_D_ = 1.3 µM)^12^. Since then, numerous studies have confirmed that neutralizing antibodies and nanobodies from Covid-19 patients also bind primarily to the RBD^13,14^, solidifying the spike protein as a target for both detection and therapeutic efforts.

Peptides present an interesting opportunity in the development of new point-of-care diagnostics against SARS-CoV-2. Peptides have been used previously for the detection of viral surface proteins and their low production cost, superior stability, high target affinity, small size, and ease of modification make them attractive in the anti-viral space^15^. The availability of mature in silico tools to model three-dimensional protein interactions provides a platform to rapidly design and optimize potential binding sequences for a specific target protein. These tools have been used to identify miniproteins capable of blocking the interaction between SARS-CoV-2 RBD and ACE2^16^. Computational approaches to peptide design have also been employed to design peptide binders with higher predicted SARS-CoV-2 RBD affinity than the ACE2-derived SBP1^17,18^, however a lack of simple and high-throughput experimental platforms for testing these peptides has, for the most part, prevented their validation in vitro. One approach taken to bridge this gap has been the application of a combinatorial affinity selection—mass spectrometry platform to screen and identify peptide binders with nanomolar affinity to SARS-CoV-2 RBD^19^. Peptide microarrays offer an alternative approach for rapid screening of large peptide libraries. Custom arrays are available from commercial vendors and can be imaged on a variety of commonly available scanners without the need for specialized training or instrumentation. In response to the SARS-CoV-2 outbreak, microarray technology has been leveraged extensively. The ability to print the entire viral proteome as short overlapping peptide fragments has enabled researchers to build linear epitope landscapes of the SARS-CoV-2 proteins, rapidly identify immunoreactive epitopes, and isolate virus-specific antibodies from patient sera^20–24^. Though antibody cross-reactivity between SARS-CoV-2 and other human coronaviruses is common, several unique immunodominant epitopes of the SARC-CoV-2 spike and nucleocapsid proteins have been identified and may serve as capture antigens for future COVID-19 diagnostic antibody tests^21,22^. Albeit less common, the same microarray technology can also be adapted to screening large de novo peptide libraries for binding to protein targets. To our knowledge, the combination of computational peptide design and microarray screening has not been previously reported for viral protein targets.

Here we demonstrate the use of a rapid and high-throughput platform to experimentally screen for binding of in silico designed peptides to SARS-CoV-2 S protein using commercially available microarray technology. Identified binders were further validated with traditional ELISA and biolayer interferometry (BLI) methods and found to have nanomolar affinity for the S protein, thus confirming the utility of computational peptide-protein design and the ability of the microarray platform to identify efficient binders. Additionally, we integrated the top peptides into an electrochemical based sensor to demonstrate their real-world applicability in saliva-based antigen tests.

## Results and discussion

### Computational design of SARS-CoV-2 S protein binding peptides

Computational design of peptides requires the generation of tertiary structures in order to predict binding to a target of interest. Analysis of the neutralizing sites on the SARS-CoV-2 S protein indicated that most monoclonal antibodies (mAbs) targeted the receptor binding domain (RBD) of the S protein^25,26^, while only one targeted the N-terminal domain (NTD)^27^. Cryo-electron microscopy structure analysis of the S protein trimer revealed that the RBD can be in an open or closed state^28^. Based on structural information, Barnes et al.^26^ identified four different classes of RBD-targeting antibodies depending on whether they bound to the ACE2 binding domain, and whether the S1 RBD was in the closed or open state. Therefore, in the computational design of binding peptides we modeled three different structures of spike protein: S protein trimer in the open form (PDB ID: 6VYB), S protein trimer in the closed form (PDB ID: 6VXX), and RBD of the S protein (PDB ID: 6LZG).

Two approaches were used to generate a library of peptides. In the first approach, we used a fragment from the N-terminal α1 helix of the human ACE2 receptor, shown to be directly involved with binding to the RBD of the S protein^9,12^. Ten 18-mer peptide sequences spanning the length of the original ACE2 fragment were generated and their tertiary structures were modeled from the crystal structure of the RBD/hACE2 complex (PDB ID: 6LZG). These peptides were subsequently docked to the RBD of the spike protein to generate protein-peptide complexes for sequence optimization.

In the second approach, we designed a set of peptides from a pool of random sequences. Since short peptides are very flexible and typically lack a distinct conformation in their unbound state, an ensemble of 20 structures was generated for every peptide sequence, as described in the Methods section. These peptides were docked to the S1 subunit of the S protein trimer in its open and closed forms, as well as to the monomer RBD structure, with no constraints on the location of the binding site. Analysis of docking complexes revealed several hot spots for peptide binding on both the NTD and RBD. About 300 protein-peptide complexes (with peptides bound to each the NTD and RBD) were selected for sequence optimization.

Sequence optimization was performed as an iterative process where mutations were introduced into every peptide sequence and kept if they resulted in an improved binding score. The most important, but also most difficult, part of the proposed approach was to correctly score and rank the protein-peptide complexes based on their binding affinity. Current scoring functions use different simplifications to enable search through both the sequence and conformational space. To account for these differences, we used the consensus score of two different scoring functions (*ddg* provided by Rosetta and ZRANK^29^ developed at Boston University) to rank sequences based on predicted binding affinity to the S protein. Using the consensus scores generated by computational analysis, we selected a library of 2,376 unique peptide sequences for screening. This library consisted of 10 wild-type ACE2 variants (as shown in Fig. 2a), 800 ACE2-optimized sequences as described in approach 1, and 1566 S protein binding sequences as described in approach 2.

### Microarray screening of designed peptides

One critical capability missing from current efforts to design binding sequences against SARS-CoV-2 is the ability to test candidates in a simple and high-throughput format. Here, we applied a fast and simple microarray-based screening pipeline to select S protein binding peptides from our in silico designed library. All reagents used in this pipeline were commercially available and required no special modifications or equipment, thereby allowing for easy adoption in other laboratories. Since the S protein trimer was not commercially available at the time of screening, we screened for binding to the SARS-CoV-2 S1 subunit, which contains the receptor binding domain (RBD) and N-terminal domain (NTD)—two of the binding hot-spots identified during computer-based docking studies.

**Figure 1:**
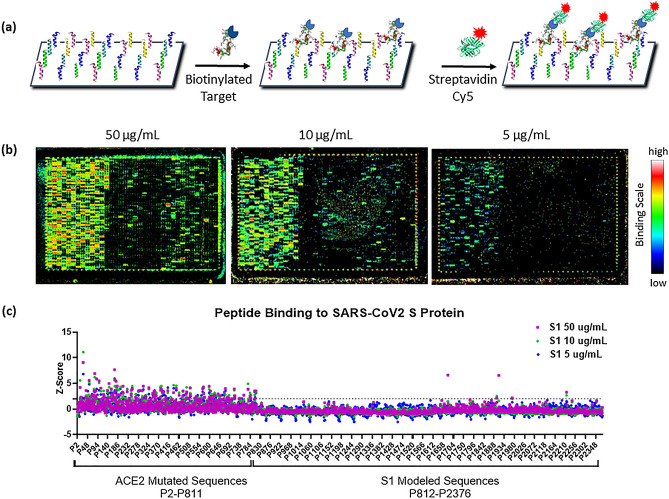
Peptide Microarray to Identify SARS-CoV-2 S Protein Binding Sequences. (**a**) Schematic showing microarray screening procedure for detecting binding of a biotinylated target protein. (**b**) Microarray images of peptide subarray following exposure to 50, 10, or 5 µg/mL of SARS-CoV2 S1 protein. (**c**) Normalized binding signal of peptides after exposure to SARS-CoV2 S1 protein at 50, 10, or 5 µg/mL concentration. Z-score at each S1 concentration is plotted for peptides listed in numerical order along the X-axis. P2-P811 correspond to peptides derived from the ACE2 N-terminal alpha helix (approach 1) while P812-P2376 were derived from a random starting library and screened in silico for docking to S protein (approach 2). Peptides with high Z-scores (> 1.95, indicated by dotted line) represent S1 protein binding sequences.

The top-ranking peptide sequences identified from in silico design were printed on a custom peptide microarray with side-by-side duplicates. The library of 2,376 sequences fit on a 1 × 2 design where two copies of the array were printed onto a single slide. Each subarray was exposed to biotinylated SARS-CoV-2 S1 protein at a single concentration between 2 and 50 µg/mL (or buffer-only control) and binding sequences were identified following incubation with streptavidin conjugated fluorescent dye, as shown in Fig. 1a.

One advantage of this system is that processing of multiple arrays can easily be completed in a single day, allowing for rapid screening of the entire library under multiple experimental conditions. Representative images of the array are shown in Fig. 1b following exposure to 50, 10, and 5 µg/mL of S1 protein. In addition to the control spots, visible as bright and dark spots around the perimeter, a large number of apparent binders are visible on the left third of the array. These spots correspond to the portion of the library (P2-P811) designed from the ACE2 receptor fragment, as described in approach 1. Not surprisingly, the design of peptides from a known binding sequence resulted in numerous “hits” with a range of binding affinities, as observed by the variation in fluorescent intensity from this region. Following data normalization, these sequences also had the highest proportion of binders with 115 of 800 sequences showing a Z-score greater than 1.95 (Fig. 1c) at the highest S1 protein concentration tested.

**Figure 2:**
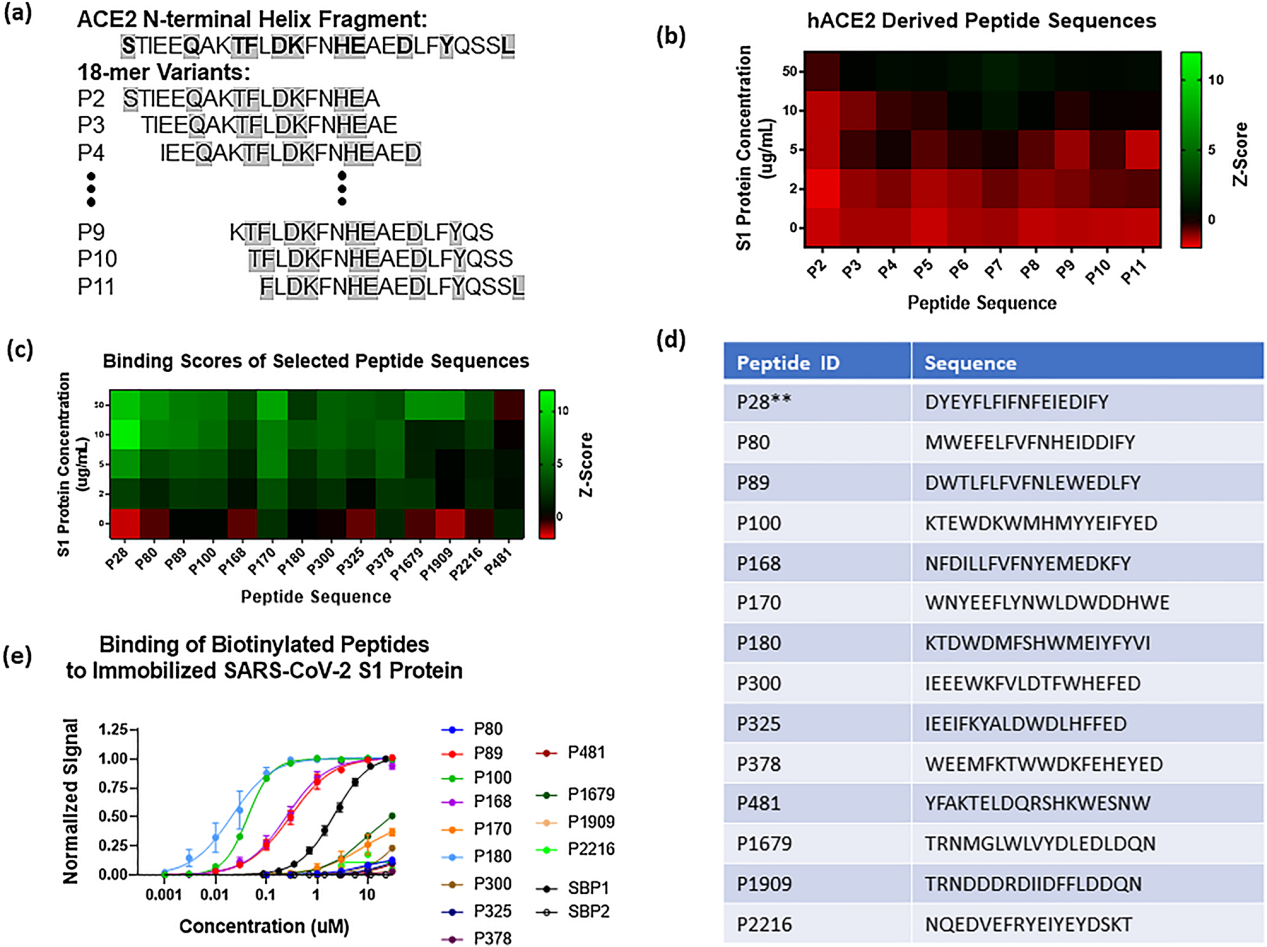
Screening and selection of peptide binders to SARS-CoV-2 S protein. (**a**) Peptides P2-P11 represent wild-type versions of the original 27-mer ACE2 N-terminal alpha helix. They were designed as 18-mers spanning the length of the original fragment with 17 amino acids overlapped. The ACE2 fragment is predicted to bind to the SARS-CoV2 S protein via residues shown in bold^12^. (**b**) Normalized binding signal of the ACE2-derived peptide variants show little to no binding to SARS-CoV2 S1 protein. There is an apparent trend for increased binding from the peptide fragments overlapping the center of the WT sequence and P7 shows the highest binding signal with a z-score of 1.3 when exposed to 50 µg/mL of S1 protein. (**c**) Normalized binding signal of the 14 peptides selected from microarray screening experiments for further characterization. 10 peptides were selected from the pool of ACE2 mutants, 3 were selected from the pool of modeled sequences, and one non-binding sequence was selected for comparison. All sequences selected had a Z-score > 2 on the 50 µg/mL S1 protein array, except for P481. (**d**) Sequences of the 14 peptides selected for synthesis with N-terminus biotin attached via a PEG4 spacer. **Note that P28 was not able to be synthesized by the vendor. (**e**) Binding curves of biotinylated peptides to immobilized SARS-CoV2 S1 protein in ELISA plate-based assay. Four peptides (P89, P100, P168 and P180) showed higher binding affinity than the original ACE2 fragment (SBP1) and were selected for further characterization.

Perhaps most interesting, however, is that the ten wild-type ACE2 variants (P2-P11), used as the starting sequences for optimization in approach 1 (Fig. 2a), showed very little binding to S1 protein. Despite the low signals, there was a trend for increased binding from peptides overlapping the central region of the original ACE2 sequence. In fact, P7, which contained 9 of the 11 binding residues present in the original fragment (Fig. 2b), showed the highest binding to S1 protein, although it never achieved a normalized binding signal greater than 1.3. The increase in binding signal obtained from sequences derived from the wild-type ACE2 variants highlights the importance of the sequence optimization process for improving target binding affinity.

Additionally, a handful of binding sequences from the pool of random sequences were observed to have high affinity to the S protein (P812-P2377, right two-thirds of array in Fig. 1b). Importantly, these sequences were obtained de novo with no inputs from a priori binding characterization. After data normalization, a total of 5 sequences were identified with a Z-score greater than 1.95 at the highest S1 protein concentration tested. Taking into consideration normalized binding signal across all S1 protein concentrations tested, we selected 14 peptides for further characterization: 10 from the pool of ACE2 mutants, 3 from the pool of S1 modeled sequences, and one non-binding sequence (P481) as a negative control for comparison. A heatmap of Z-scores for each of the selected peptides across all S1 protein concentrations tested is shown in Fig. 2c with corresponding sequences in Fig. 2d. All sequences selected had a normalized binding signal greater than 2 on the 50 µg/mL S1 protein array, except for P481 (the non-binding sequence). Importantly, not including time for array production, the entire screening process could be completed in less than one week, showing that potential binding sequences can be identified rapidly in response to emerging targets.

### Peptide binding characterization

To confirm and further characterize the binding observed on the microarray, we turned our attention to more traditional methods. We started by setting up an ELISA-like assay to measure binding of biotinylated peptides to immobilized SARS-CoV-2 S1 protein, as described in the Methods section. We validated this assay with a 23-mer control sequence (SBP1) from the N-terminal alpha helix of the ACE2 receptor, which had been shown to bind the SARS-CoV-2 S protein RBD with micromolar affinity^7^. In our ELISA-like assay, biotinylated SBP1 had a calculated K_D_ of 2.2 µM to the SARS-CoV-2 S1 protein while a 12-mer truncated version of the peptide (SBP2) showed no measurable binding (Fig. 2e). Using the same assay format, we also screened our selected peptide sequences for binding to S1 protein. Of the 14 sequences selected from the microarray, 13 were able to be synthesized with an N-terminus biotin attached via a PEG4 spacer (Fig. 2d). Testing of the peptides in the assay identified four lead candidates with stronger binding to the S1 protein compared to the benchmark peptide, SBP1 (Fig. 2e). As expected, P481 showed no measurable binding signal to S1 protein. Notably, all four of the lead peptide binders were from the pool of ACE2 derived sequences.

**Figure 3:**
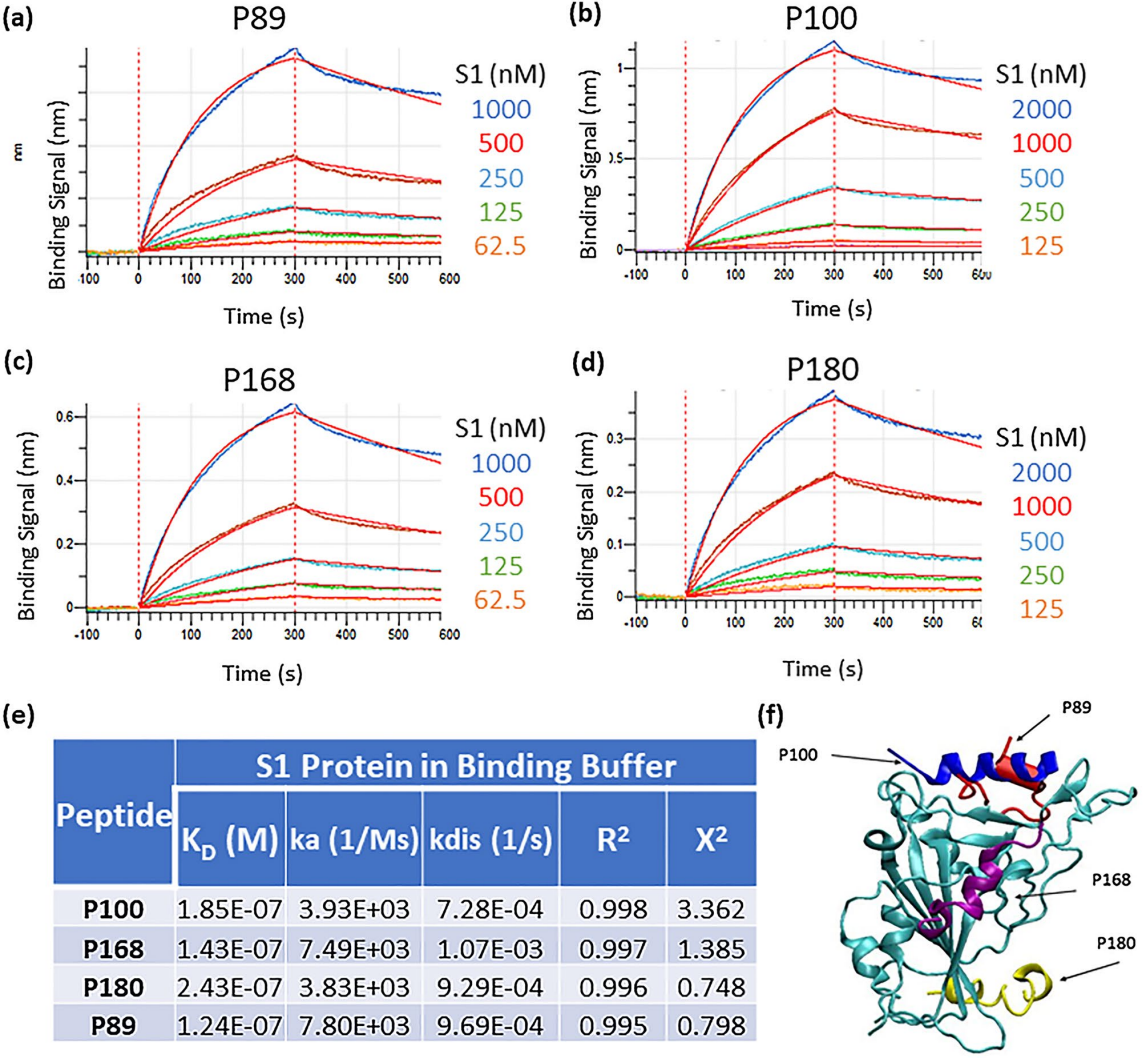
Binding characterization of peptide binders. (**a**–**d**) BLI binding traces of S1 protein association and dissociation to each of the top four biotinylated peptides (immobilized onto streptavidin sensors). (**e**) The calculated binding constants for the top four peptides. (**f**) Potential binding sites for the top-4 peptides on the RBD subunit of the spike protein (colored in cyan) as identified from the docking simulations. Peptides P89 (red) and P100 (blue) bind to the ACE2 binding site, peptide P180 (yellow) binds to the same site as CR3022 and S2A4 antibodies, while the binding site for peptide P168 (purple) is located similarly to the antibody S309 binding site^25,30–32^.

Next, we used bio-layer interferometry (BLI) to further characterize the binding interactions between our top four peptide candidates and the S1 protein. This assay allows for the measurement of kinetic parameters and the immobilization strategy is more relevant to our endpoint sensing platform (described below). Here, biotinylated peptides were immobilized onto streptavidin coated biosensor tips and dipped into wells containing free S1 protein in solution. The presence of the biotin tag on the N-terminus of the peptide sequence forces the peptide into a specific orientation when immobilized, which may limit accessibility to the target protein, but is required for use as a diagnostic. The association and dissociation curves from serial dilutions of the S1 protein are shown for each of the peptides in Fig. 3a–d. After global 1:1 curve fitting, the dissociation constants (K_D_) for all peptides were determined to be between 100 and 250 nM (Fig. 3e), with P89 having the highest affinity (K_D_ = 124 nM). While the top 4 peptides identified in this study were selected for binding to SARS-CoV-2 S protein, preliminary selectivity screening against other viral proteins indicated that some cross-reactivity may be present and needs to be further evaluated (Supplemental Fig. S1).

We also performed computer modeling to identify the potential binding sites of these peptides on the SARS-COV-2 S protein. The 3D structures of the peptide sequences were generated using the Rosetta package and peptide docking to the RBD was performed using the ZDOCK, as described in the Methods section. As shown in Fig. 3f, the predicted binding site for peptides P89 and P100 encompasses residues Y449–Q493 and overlaps with the ACE2-binding site^32^. Potential binding sites for the two other peptides are located outside of the ACE2-binding region. Peptide P180 binds to the residues Y380–F392 of the S protein, which was identified as the binding site for the S304 and CR3022 antibodies^31^, while P168 binds to residues E340–K356 of the RBD, consistent with the binding site of the S309 antibody.

### Electrochemical impedance spectroscopy (EIS) for detection of SARS-CoV-2 S protein

**Figure 4:**
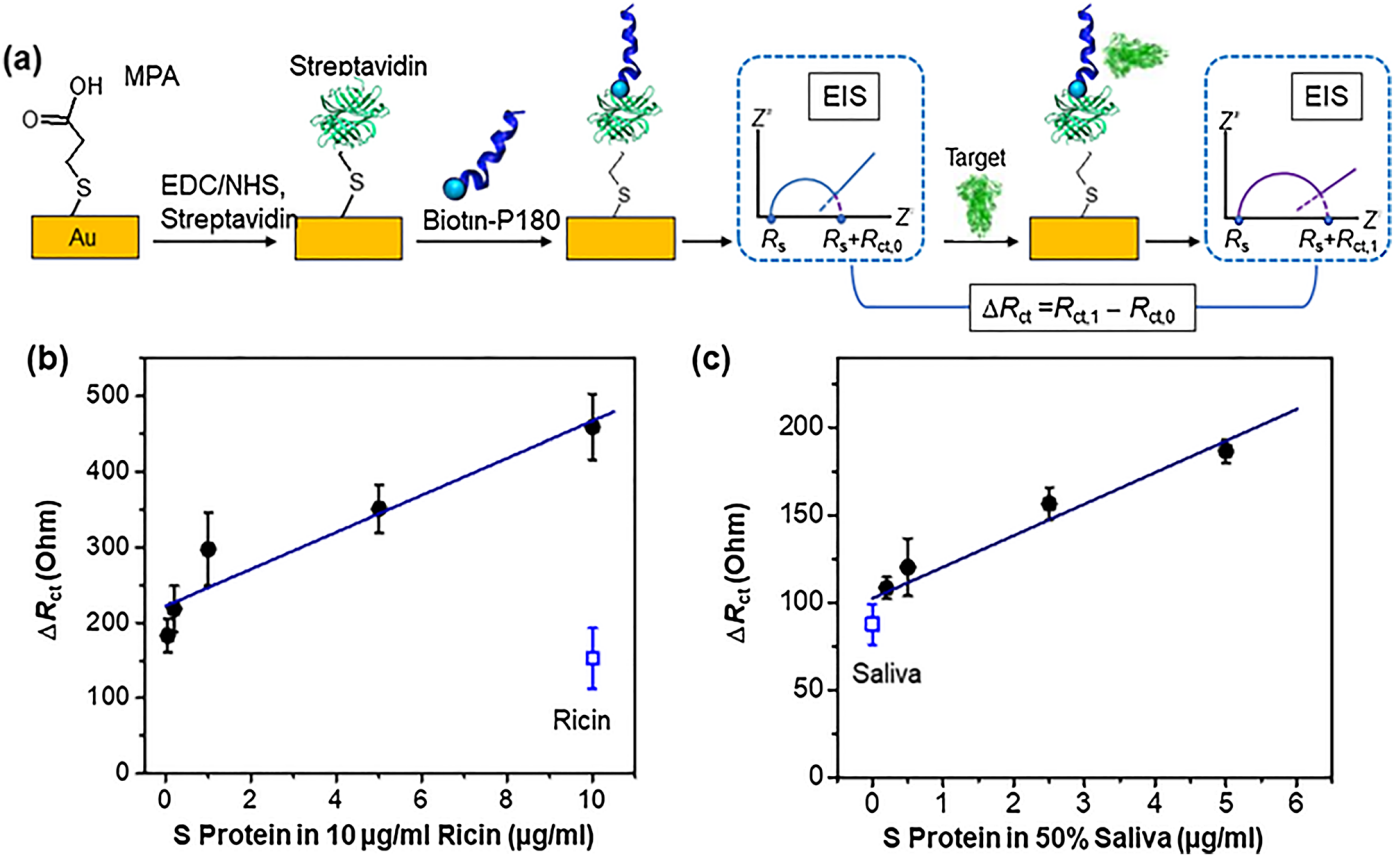
Electrochemical sensing of s protein with peptide binder. (**a**) Schematic of the immobilization of the P180 peptide on gold electrodes for EIS measurements to quantify binding with SARS COV2 S protein based on alteration in charge transfer resistance (DR_ct_). (**b**) Signal (DR_ct_) as a function of S protein levels in Ricin background at 10 µg/mL. (**c**) Signal (DR_ct_) as a function of S protein levels in 50% saliva.

To demonstrate the application of these peptides as the sensing element for the detection of SARS-CoV-2 S protein, we integrated them into an electrochemical-based sensor to detect S protein from spiked saliva. The peptides were immobilized on self-assembled monolayer modified gold electrodes and alterations in the charge transfer resistance (ΔRct) were used to quantify the binding signal by electrochemical impedance spectroscopy (EIS). Results for peptide P180 are shown in Fig. 4a (detailed results in Supplemental Fig. S2). EIS results were obtained in phosphate buffer solution (0.1 M, pH 7.2) containing 10 mM K_3_[Fe(CN)_6_]/K_4_[Fe(CN)_6_], with the frequency scanned from 100 kHz to 0.1 Hz at the potential of the redox probe (0.135 V vs. Ag/AgCl). Charge transfer resistance (Rct) values were obtained by fitting the EIS spectra using Randell circuit model provided by Solartron. The plot in Fig. 4b of the signal (ΔRct) for varying S protein levels (0.05–10 µg/mL) that are present in a background of 10 µg/mL Ricin as the non-specific control protein shows a steady rise in signal due to specific binding to the S protein that leads to progressive blockage of the electron transfer. To show the potential of this sensor platform to function in saliva matrices, the S-protein was spiked at varying levels into 50% saliva. The plot in Fig. 4c shows a linear rise in the signal (ΔRct) as a function of S protein concentration in saliva. We assess the limit of detection for this sensor as ~ 0.1 µg/mL in phosphate-buffered saline (PBS) and ~ 0.2 µg/mL in saliva matrices, based on signals that are at least three standard deviations above a negative control protein (10 µg/mL Ricin in PBS, Fig. 4b) and no target in saliva (Fig. 4c). We are currently developing alternate sensor paradigms based on nanoporous gold electrodes^33^ and redox signal amplification to improve sensitivity^34^, however, the current limit of detection would be relevant for detection of SARS-CoV-2 in clinical samples^35,36^.

## Conclusions

The ability to rapidly develop diagnostic and therapeutic agents to address emergent viral threats would provide the means to monitor and prevent the spread of a disease, protecting the most vulnerable population. In this work, we developed a rapid pipeline to design peptide sequences with the potential to bind the S protein using computational methods and a simple and fast screening method based on peptide microarray technology. The screening and data analysis to identify peptide binder candidates takes less than a week to be executed and is based on commercially available instrumentation and materials. Finally, we demonstrated that the microarray platform, which mimics most sensor platforms in which a bio-recognition element is anchored to a surface, is capable of identifying peptide sequences that can be rapidly integrated in an electrochemical sensor with a clinically relevant limit of detection. We believe the main advantage of this approach is the general applicability to other targets of interest. Depending on the emerging threat landscape, we believe that SARS-CoV-2 variants or even completely unrelated viruses could be addressed. Furthermore, our lab is currently working to expand the pipeline for selection of peptide BREs for a wider variety of targets of interest to the Department of Defense. We acknowledge there are still some limitations that need to be addressed. Mainly (i) the time required for computational design, (ii) the need for a crystal structure for the target protein, (iii) the limited length of peptides that can be synthesized on the array (18-mers), and (iv) the time to receive the arrays after ordering (4–6 weeks). As structure prediction models continue to evolve, we expect the computational component of this research to become much faster and less reliant on the availability of crystal structures. The knowledge of existing binding epitopes on target proteins greatly increases the chance of success during the computational design and sequence optimization stage, resulting in the identification of efficient binders in a single round of screening. We acknowledge that current peptide microarray technology limits the ability to test longer sequences, which may provide more complex structures with better binding affinities. Shorter peptides have been shown capable of achieving high affinity binding but may require multiple rounds of optimization, thereby extending the time required for candidate selection due to lengthy array printing time. We believe that further advances in microarray technologies will overcome these issues providing a more versatile option for peptide design and testing to address new viral threats in a timely fashion.

## Materials and methods

### Computer modeling

Two methods were explored to generate a peptide library for the SARS-CoV-2 S1 protein. In the first we used a 27-mer peptide fragment derived from the N-terminal alpha-helix of the human ACE2 receptor, shown to be in direct contact with the SARS-CoV-2 S protein RBD during binding of viral particles. The sequence of this fragment was split into ten overlapping 18-mer peptide sequences and tertiary structures were predicted from the crystal structure of the RBD/hACE2 complex (PDB ID: 6LZG). These peptides were subsequently docked to the S protein and used as the initial set of protein-peptide complexes for sequence optimization.

In the second approach, we began with a random 18-mer peptide sequence. An ensemble of 3D structures was generated for every sequence to account for their flexibility. Initially, 1000 s of tertiary structure models for the target sequence were created using the *AbinitioRelax* protocol from the Rosetta package^37^. The models were clustered and structures from five clusters with the lowest energy were selected as input structures for the replica exchange Molecular Dynamics (REMD) simulations^29^. The REMD simulations were performed with the Amber16 suite of biomolecular simulation programs^20,38^. Eight replicas distributed over a temperature range from 270 to 600 K were used. The implicit water simulations for each replica were initially equilibrated for 200 ps at the corresponding temperature followed by 5 ns of production run. The time step was set to 2 fs and SHAKE was applied to constrain the bonds connecting hydrogen atoms. The temperature exchanges were attempted every 1 ps and 500 snapshots from the production run were used for cluster analysis. Clustering was performed with the *Cpptraj* module based on the pairwise backbone-atom only root-mean square deviations (RMSD). Saved snapshot conformations were clustered into ten clusters and a representative from four clusters with the lowest energy were taken for the docking. A total of 20 structures for every peptide sequence were used in the docking calculations.

The rigid docking of the initial peptides to the spike protein was performed using the ZDOCK package developed at Boston University^39^. Both protein and peptides were considered as rigid and a global search of the rotational and translational space was performed without any constraint on the location of the binding sites. For every peptide structure, the program generated and clustered 2000 docking conformations from which the top 4 protein-peptide complexes were selected for sequence optimization with the Rosetta package.

Peptide sequence optimization used the Monte Carlo-based RosettaScripts protocol that included a random single base mutation, side chain rotamer repacking, and backbone and side chain torsion minimization. The scoring metrics were calculated using Rosetta’s scoring functions: binding energy of the complex *ddg* and interface buried solvent accessible surface area *sasa*. The mutation was accepted if the scoring metrics of the mutated sequence were better than the starting sequence. For every protein-peptide complex input, the program screened 10,000 peptide sequence mutants in order to generate the top 10 protein-peptide complexes with optimized binding.

Two different scoring functions were used to rank the generated complexes: *ddg* scoring function provided by the Rosetta package and ZRANK scoring function^29^ developed at Boston University. The designed protein-peptide complexes were ranked using consensus score and peptide sequences with the highest scores were selected and processed for microarray screening.

### Microarray screening

The library of 2,376 sequences was printed onto a custom PEPperCHIP peptide microarray (PEPperPrint GmbH, Heidelberg, Germany). 18-mers were printed in numerical order with side-by-side duplicates. Positive (hemagglutinin (HA)) and negative (G residue) control sequences were printed around the perimeter of the array for quality control assessment and grid alignment. Two copies of each array were printed on a single microarray slide. To screen for protein binding, each array was blocked (Rockland Blocking Buffer) for 1 h and then incubated with biotinylated SARS-CoV-2 S1 protein, (SARS-CoV-2 S1, His, Avitag #S1N-C82E8, Acro Biosystems, Newark, DE) in binding buffer (1 × PBS + 0.05% tween20 + 0.5% BSA) for 1 h. Between each step the array was washed 3 times for 1 min each in wash buffer (1 × PBS + 0.05% tween20) to remove any non-specific protein binding. Binding of biotinylated protein was detected by incubating the array with streptavidin conjugated to Cy5 dye (SA-Cy5, 1:20 dilution) while control spots were detected with an anti-HA antibody conjugated to Cy5 dye (anti-HA-Cy5, 1:2000 dilution, PEPperPRINT). Both were added in binding buffer for 30 min. The array was scanned on an Agilent SureScan microarray scanner in the red channel (640 nm) with 3 µm resolution and 20-bit dynamic range. Tiff image files were loaded into image analysis software (Mapix v8.5 by Innopsys), overlaid with grid file (.gal) to designate spot location, and median fluorescence intensity for each spot was extracted and averaged across replicates. Spots with visual artifacts (scratches, dust, etc.) were excluded from analysis. In order to compare binding intensities across different arrays and account for experimental variations in signal, sequence intensities were converted into Z-scores, using Eq. (1) shown below^20,38^:

1 $$Z{-}score=({intensity}_{P}-{mean\,intensity}_{{P}_{1}\ldots {P}_{n}})/{SD}_{{P}_{1}\ldots {P}_{n}}$$

where P is any peptide sequence on the array and P1…Pn represents the aggregate measure of all peptides on the array.

### Peptide synthesis

Selected peptides were synthesized by a commercial vendor (Peptide2.0) with an N-terminal biotin attached by a PEG4 spacer and HPLC purified to achieve > 98% reported purity. Peptides were resuspended in 100% DMSO and diluted into PBS to achieve a stock concentration of 1 mM. Sonication and gentle warming were used to improve solubility in the event the peptides precipitated during dilution.

### Peptide binding validation with ELISA

An in-house ELISA-like assay was developed to measure binding of biotinylated peptides to immobilized SARS-CoV-2 S1 protein (His-tag, #S1N-C52H3, Acro Biosystems). Briefly, proteins diluted in 1 × PBS were adsorbed (0.2 µg per well) to a 96-well plate overnight at 4 °C. The following day, the plate was blocked (Blocking Buffer, Rockland) for 1 h at RT. The plate was washed 3 times with wash buffer in between each step. Biotinylated peptides were diluted in binding buffer and incubated on the plate, in duplicate, at RT for 1 h. Binding was detected with streptavidin-HRP (1:200 in binding buffer) followed by TMB substrate for colorimetric development. Signal development was stopped with the addition of 2 M sulfuric acid and absorbance values were read on a SpectraMax Paradigm plate reader (450 nm, Molecular Devices). Raw absorbance values were normalized to MIN and MAX wells on each plate. Binding curves were graphed in GraphPad Prism (v8.4) and binding constants (K_D_) estimated with non-linear curve fitting (one-site specific binding with hill slope). Data points represent the mean and standard deviation of replicate wells.

### Binding characterization using bio layer interferometry (BLI)

A ForteBio Octet RED96e Bio-Layer Interferometry system (ForteBio, CA) was used to characterize peptide-protein binding kinetics for each of the top four SARS-CoV-2 S protein binding peptides. All reactions were completed at 30 °C and 1000 rpm. Biotinylated peptides were immobilized onto pre-hydrated streptavidin (SA) biosensor tips (Forte Bio). Peptide loading time and concentration was optimized to obtain optimal loading density (as previously described^39^). Tips loaded with peptide were baselined in an appropriate buffer (matched to protein solution), and then dipped into wells containing SARS-CoV-2 S1 protein (His-tag, #S1N-C52H3, Acro Biosystems) serially diluted in binding buffer to obtain the association constant (K_a_). Double referencing was completed by subtracting off background signal from a peptide only sensor (no protein) and reference sensors (no peptide) to account for non-specific interactions of protein. After association, the tips were dipped back into the baseline well to obtain the dissociation constant (K_dis_). Curve fitting was completed with ForteBio Biosystems using a global fitting algorithm and 1:1 binding model to obtain dissociation constants (K_D_).

### Disclaimer

The views and opinions presented herein are those of the authors and do not necessarily represent the views of the US Department of Defense or its Components.

## Supplementary Information

Supplementary Information.

## Data Availability

The datasets generated during and/or analyzed during the current study are available from the corresponding author on reasonable request.
