# Supplementary material for: A high-throughput pipeline for design and selection of peptides targeting the SARS-Cov-2 Spike protein
Source: Sci Rep. 2021 Nov 5;11:21768. doi: 10.1038/s41598-021-01225-2 (PMC8571316; doi:10.1038/s41598-021-01225-2)
Supplement: Supplementary file 1 — Supplementary Information. [file 41598_2021_1225_MOESM1_ESM.pdf]

A High-Throughput Pipeline for Design and Selection of Peptides Targeting the SARS-Cov-2 Spike Protein

Monica Wolfe[1,2], Sean Webb[1,2], Yaroslav Chushak[1,3], Rachel Krabacher[1#], Yi Liu[4], Nathan Swami[4], Svetlana Harbaugh[1], and Jorge Chavez[1*]

1) 711th Human Performance Wing, Air Force Research Laboratory, Wright Patterson Airforce Base, OH, United States; 2) UES, Inc. Dayton, OH, United States; 3) Henry M. Jackson Foundation, Dayton, OH, United States; 4) Department of Electrical and Computer Engineering, University of Virginia, Charlottesville, VA, #Current address: Materials & Manufacturing Directorate, Air Force Research Laboratory, Wright Patterson Air Force Base, OH, United States.

Supplemental Information

Peptide Binding Specificity with ELISA

An in-house ELISA-like assay was developed to measure binding of biotinylated peptides to immobilized protein (SARS-CoV2-S1, Influenza HA, or chemically-inactivated Ricin toxoid). Briefly, proteins diluted in 1x PBS were adsorbed (0.2 µg per well) to a 96-well plate overnight at 4 °C. The following day, the plate was blocked (Blocking Buffer, Rockland) for 1 hour at RT. The plate was washed 3 times with wash buffer in between each step. Biotinylated peptides were diluted in binding buffer and incubated on the plate, in duplicate, at RT for 1 hour. Binding was detected with streptavidin-HRP (1:200 in binding buffer) followed by TMB substrate for colorimetric development. Signal development was stopped with the addition of 2M sulfuric acid and absorbance values were read on a SpectraMax Paradigm plate reader (450 nm, Molecular Devices). Raw absorbance values were normalized to MIN and MAX wells on each plate. Binding curves were graphed in GraphPad Prism (v8.4) and binding constants (K_D) estimated with non-linear curve fitting (one-site specific binding with hill slope). Data points represent the mean and standard deviation of replicate wells.

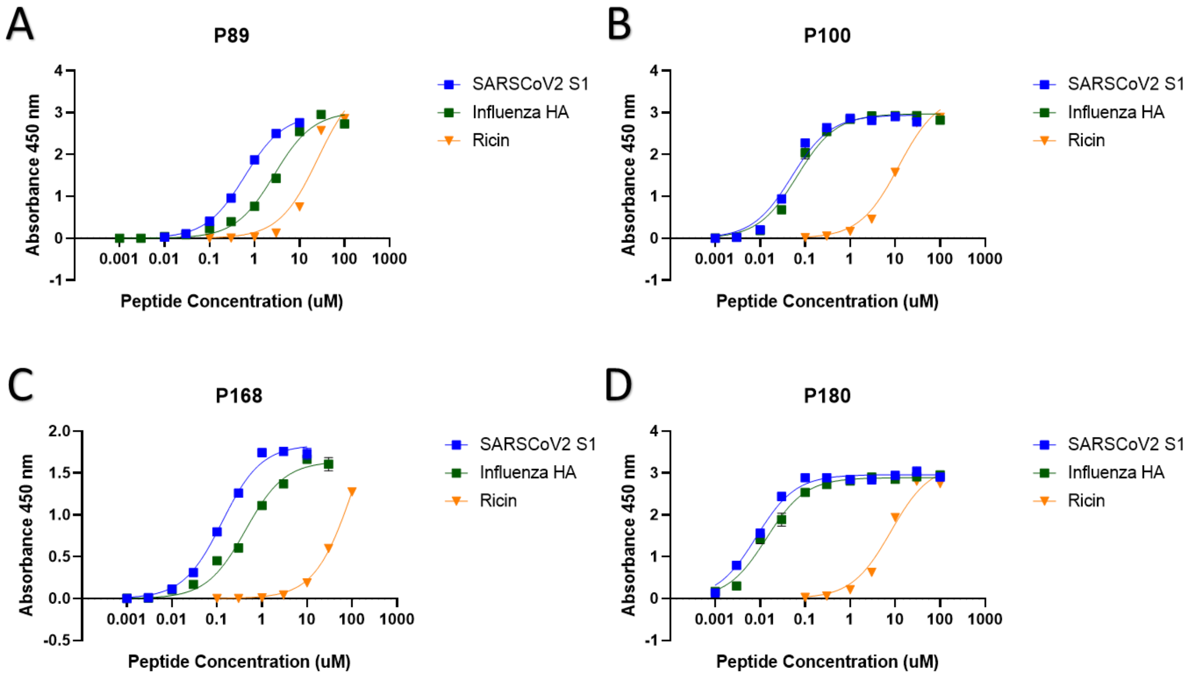

E

Peptide	Binding Constant (Kd)		
	S1 (uM)	HA (uM)	Ricin (uM)
**P100**	0.05	0.066	11.93
**P168**	0.13	0.432	112.1
**P180**	0.009	0.014	7.983
**P89**	0.55	2.7	25.66
**SBP1**	13.4	6.9	98

Figure S1. Binding curves for P89 (A), P100 (B), P168 (C) and P180 (D) in ELISA-like assay with SARS-CoV-2, Influenza HA, or Ricin proteins to assess selectivity of binding. Estimated binding constants (E) indicate some level of cross-reactivity with Influenza HA protein.

Preparation of electrodes for EIS Sensor

The gold electrodes (DropSens, Spain) were cleaned by potential cycling in 0.1 M H_2SO_4 between -0.4 and +1.5 V at a rate of 0.1 V/s for 30 cycles. The cleaned electrodes were soaked in 10 mM 3-mercaptopropionic acid (MPA) solution overnight to form MPA covered gold. Then the MPA-gold surface was activated by incubating for 1 h in 10 mM 2-(N-morpholino) ethanesulfonic acid (MES) buffer solution (pH 5.2) containing 1 mM N-(3-Dimethylaminopropyl)-N′-ethylcarbodiimide (EDC) and 4 mM N-Hydroxysulfosuccinimide (NHS). Streptavidin was conjugated on the activated surface by incubating in streptavidin (0.01 mg/ml in 10 mM PBS) overnight at 4 °C, followed by thorough rinsing in PBS containing 0.05% Tween 20 (PBST) and DI water. Biotinylated peptides (0.01 mg/mL in 10 mM PBS) were conjugated on the streptavidin coated surface by incubating for 3 h, followed by thorough rinsing with PBST and DI water. After interrogating the peptide covered surface with EIS, the electrodes were exposed to SARS-CoV-2 S protein (His-tag, #S1N-C52H3, Acro Biosystems) in solution (either in 10 µg/ml Ricin in PBS or 50% saliva/PBS) for 30 min at room temperature. Ricin toxoid, Chemically Inactivated from Ricinus communis (#NR-4671, acquired from BEI Resources, NIAID, NIH) was used as a negative control for non-specific protein binding. Pooled human saliva was acquired from Innovative Research (#IRHUSL, Innovative Research, MI, USA). After rinsing with PBST and DI water, the electrodes were again interrogated with EIS. Representative EIS spectra (**Figure S2**) show progressive increase of charge transfer after each surface modification, suggesting successful binding of the corresponding molecules on gold surface.

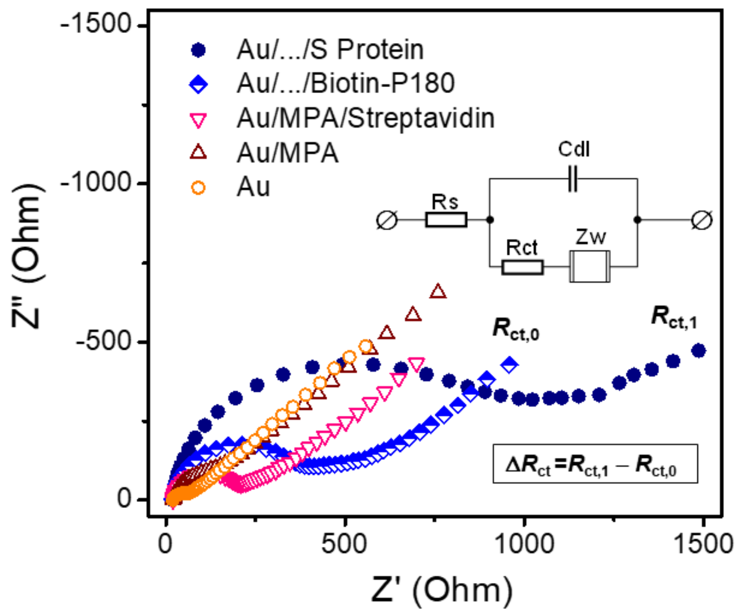

Figure S2. EIS of gold electrode after surface modification.
